# *Xylosandrus germanus* (Blandford, 1894) on Grapevines in Italy with a Compilation of World Scolytine Weevils Developing on Vitaceae

**DOI:** 10.3390/insects12100869

**Published:** 2021-09-24

**Authors:** Enrico Ruzzier, Stefan Cristian Prazaru, Massimo Faccoli, Carlo Duso

**Affiliations:** Department of Agronomy, Food, Natural Resources, Animals and Environment, University of Padova, Viale dell’ Università 16, 35020 Legnaro, Italy; stefancristian.prazaru@studenti.unipd.it (S.C.P.); massimo.faccoli@unipd.it (M.F.); carlo.duso@unipd.it (C.D.)

**Keywords:** ambrosia beetles, grapevine, cv Glera, invasive alien pests

## Abstract

**Simple Summary:**

*Xylosandrus germanus* (Curculionidae: Scolytinae: Xyleborini) has been recorded attacking grapevines (cv Glera) in the Conegliano Valdobbiadene DOCG area (Vidor, Veneto, Northern Italy). Here, we documented the first case of this invasive species damaging *Vitis vinifera* in Italy, supplying the second record ever in Europe. The type of the attack is illustrated and the possible causes of the onset of the infestation are discussed. In addition, an updated checklist of world Scolytinae attacking Vitaceae is provided. A total of 34 scolytine species, in eight tribes and fifteen genera, are recorded.

**Abstract:**

The invasive ambrosia beetle *Xylosandrus germanus* (Curculionidae: Scolytinae: Xyleborini) is recorded for the first time infesting wine grapes in Italy. The type of the attack is illustrated and the possible causes of the onset of the infestation are discussed. Furthermore, given the continuously increasing number of alien wood-borer beetles introduced worldwide, we provide and discuss the updated world checklist of Scolytinae attacking Vitaceae, and *Vitis* sp. in particular.

## 1. Introduction

Wood-boring insects can cause substantial damage to cultivated crops, particularly fruit and ornamental trees [1]. Coleoptera and Lepidoptera are the orders hosting the highest number of wood-boring species developing on Vitaceae Juss., 1789, with many species recognized as pests on *Vitis* spp. worldwide (e.g., [2,3,4,5,6,7,8,9,10]).

The tribe Xyleborini (Coleoptera: Curculionidae), with more than 1200 described species, is the largest within Scolytinae [11] and includes several invasive species worldwide (e.g., [12,13,14]), which are causing serious damage to forest ecosystems and agricultural crop systems [15].

*Xylosandrus germanus* (Blandford, 1894), also known as the black stem borer, is a xyleborine beetle native to the Eastern Palearctic and Oriental region [16] that is now widely established in North America [13], Europe [17] and Oceania [18]. It is a well-founded opinion that *X. germanus* was introduced and spread outside its territory of origin through timber trade and plants-for-planting [19]. This species may attack more than 200 species among plants and trees [20]. The attack consists of the excavation by a single foundress of an entrance tunnel, which is enlarged forming brood chambers with branch tunnels [21,22,23]. During the first days of the wood colonization, the maternal-boring activity is usually easily recognizable due to the emission from the entrance hole of a white frass cylinder [19,24]. The entire development of the offspring takes place in the chamber and each nest can host multiple individuals at different life stages; insect development takes approximately 30 days and the number of generations per year can vary between two and three depending on the environmental conditions [25,26,27].

The reproductive success of *X. germanus* depends on the ability to establish in the brood chamber a culture of *Ambrosiella grosmanniae* Mayers, McNew and Harrington, 2015 (Sordariomycetes: Microascales: Ceratocystidaceae) [28], its principal mutualistic fungus and main nourishment for both larvae and adults [29]. *Ambrosiella grosmanniae*, together with other symbiotic fungi, such as *Fusarium* sp. and *Geosmithia* sp. [30], are the cause of a defense response in the infected plants; their action contributes to the initiation of wilting, dieback, tree decline and death [31,32].

*Xylosandrus germanus* was first recorded in Italy in 1992 (Lombardy) and then again in 1993 (Piedmont), though these records were not published until 2000 [33]. In 1998, this species was next observed in Friuli-Venezia Giulia, in walnut and apple plantations [34,35]. Subsequently, it was recorded in multiple localities in Veneto [36] and Trentino-Alto Adige [37]. Recently, *X. germanus* was also collected in central Italy, in the Viterbo province and the Circeo National Park in 2018, suggesting its spread southward across the Italian peninsula [24].

*Xylosandrus germanus* was reported as a pest on grapevine for the first time in 1932, while infesting stems of vines in Long Island (New York, NY, USA) [38]. In 2003, the species was documented as causing substantial damage in Maindreieck (Germany) [39], and subsequently, in 2007, it caused severe and extensive infestations in Chengdu (SE China) [30].

In September 2019, a severe, although localized, infestation occurred in the Vidor Municipality (Treviso Province, NE Italy) in the Conegliano-Valdobbiadene Prosecco DOCG area, causing severe stress to grapevines. This event, which represents the first case of *X. germanus* attacking *V. vinifera* in Italy, is here presented, and the possible origins of this infestation are discussed.

Furthermore, given the large phytosanitary interest in scolytine as invasive alien species, the information available on bark and ambrosia beetles developing on Vitaceae are here summarized, providing a full species list of species and their host plants.

## 2. Materials and Methods

### 2.1. Study Area

**Figure 1:**
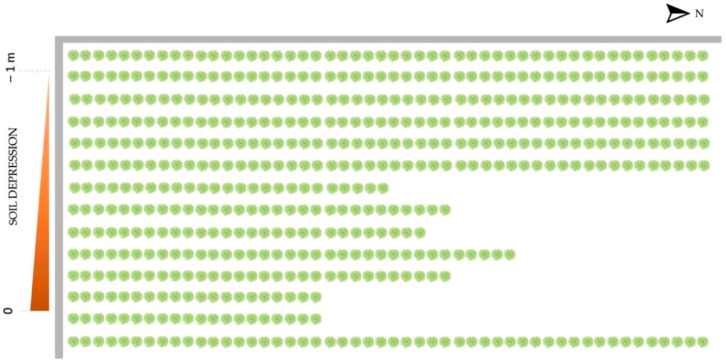
Schematic representation of the vineyard investigated (view from above). In green, the rows of grapevines; in gray, the concrete walls delimiting the field.

The infestation occurred in a small vineyard located in the Vidor Municipality (Treviso Province, Veneto Region, Italy), inside the Conegliano Valdobbiadene Prosecco DOCG area. The field, with a size of approximately 1700 m^3^, contains 809 grapevines (cv Glera) distributed along 14 rows (Figure 1). The vineyard is oriented South to North and is characterized by land sloping from the east to the west. Additionally, the field is bordered by a concrete boundary wall on its south and west sides (Figure 1).

### 2.2. Specimen Collection and Spatial Data Analysis

The infested field was investigated in September 2019, a few days after the onset of the attack. Scolytine specimens were collected directly from grapevine trunks using soft forceps, and subsequently identified using the key provided by Faccoli [40]. Infested grapevines were marked on each row; four trunks were removed and opened in the laboratory to facilitate the images.

Photographs were taken using a Panasonic Lumix DMC-FZ200 camera equipped with a Lumix DMW-LC55 lens.

Data from grape plants in the field, along with the infestation intensity, were analyzed using the SADIE ‘red-blue plots’ methodology to detect spatial patterns in the symptomatic grapevines [41]. Infestation intensity was defined as the occurrence of attacked grapes on three contiguous grape plants per row for three rows (thus creating a squared plot of 3 × 3 plants).

### 2.3. Literature Review

To compile and then review exhaustive literature on Vitaceae-related Scolytinae, we performed a careful search on Google Scholar and Scopus through the use of the keywords “Vitaceae”, “*Vitis*”, “Scolytinae” and “pest”, integrated with the usage of the Boolean operators AND, OR and NOT, and with the use of quotes for specific word combinations.

The tribes, genera and species provided in the results are listed alphabetically. The host plant taxonomy follows World Flora Online: A Project of the World Flora Online Consortium [42].

### 2.4. Species Distribution

The species distribution is based on the Catalog of Scolytidae (Coleoptera) IV published by Bright [43], integrated with records contained in publications subsequent to 2019. The distribution provided after each species name in Section 3.2 follows the system and acronyms adopted in Bright [43].

## 3. Results

### 3.1. Infestation Symptoms and Attack Incidence

**Figure 2:**
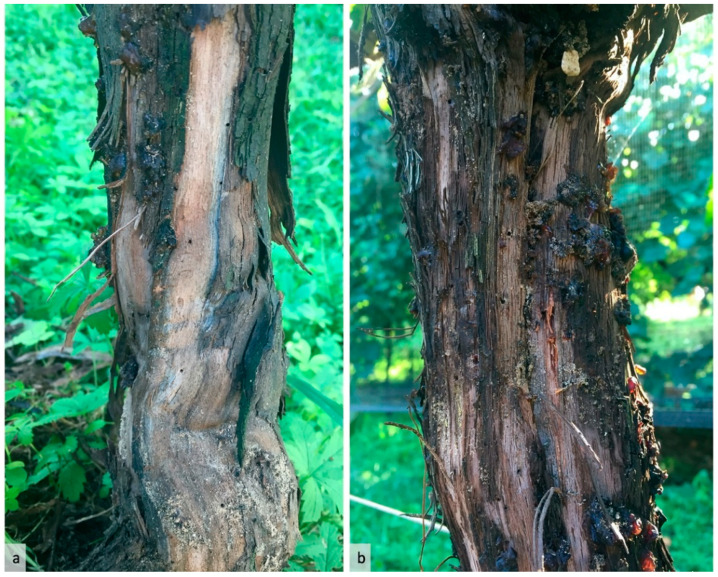
Infestation of *Xylosandrus germanus* on *Vitis vinifera*: tunneling concentrated in the proximity of the graft union (**a**) and at the head of the grapevine (**b**).

In mid-September 2019, several grapevine plants showed serious symptoms of decline, such as loss of grapes, leaf staining and defoliation. Careful inspection of such plants revealed a massive attack by *X. germanus*, with abundant tunneling along the entire trunk of the infested plants and copious emission of white frass cylinders (“noodles”) from the penetration holes. Infested plants showed a large number of tunnels concentrated near the graft union (Figure 2a) and the head of the vine (Figure 2b); no attack was observed on cordons.

**Figure 3:**
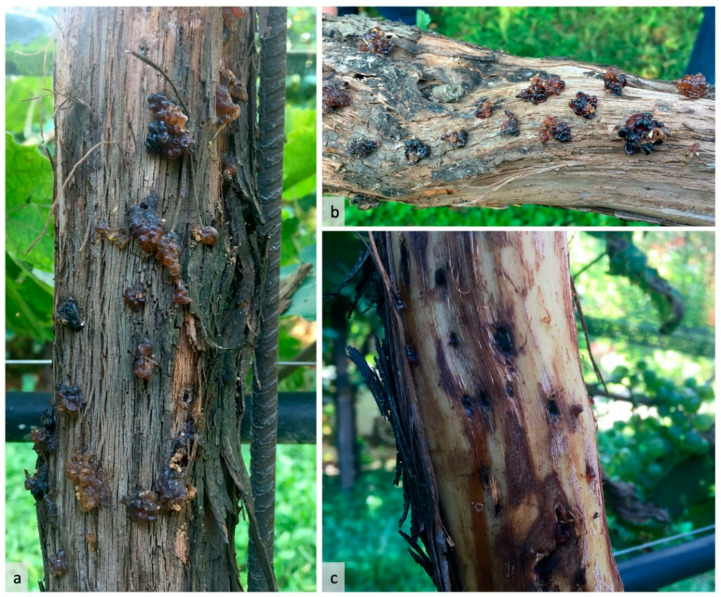
Infestation of *Xylosandrus germanus* on *Vitis vinifera* associated with the emission of gum (**a**,**b**). Decorticated grapevine presenting dark brown-black staining of the wood in correspondence to the tunnels of *X. germanus* (**c**).

A peculiarity of the attack was the abundant secretion of gum by the plant, with the consequent formation of yellow-brown concretions (Figure 3a,b). After decortication, the stems of the infested vines showed a typical brown-black staining of the wood around each entrance hole (Figure 3c).

**Figure 4:**
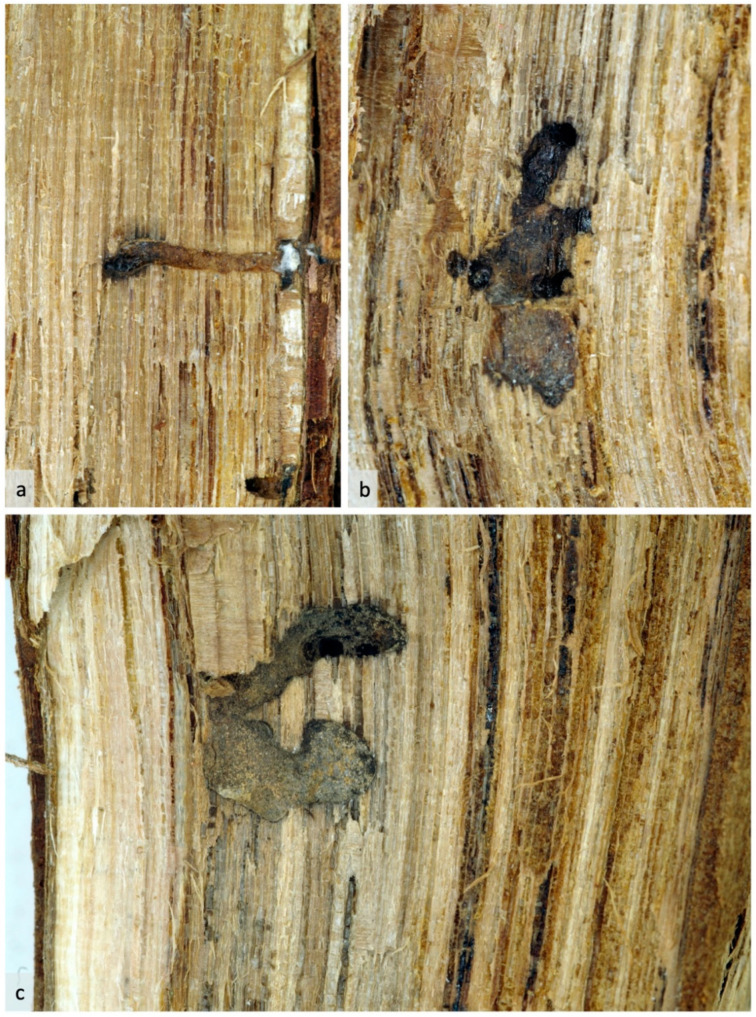
Grapevine longitudinal sections illustrating *X. germanus* galleries’ development and wood staining due to its symbiotic fungi; settlement tunnel (**a**) and rearing chambers (**b**,**c**).

Once dissected longitudinally, infested trunks showed the *Xylosandrus* tunnels with several early settlement tunnels (Figure 4a) and more complex structures with secondary tunnels and rearing chambers (Figure 4b,c). The dark color of the inner walls of the brood chambers suggested the successful establishment of the symbiont fungus *A. grosmanniae*.

**Figure 5:**
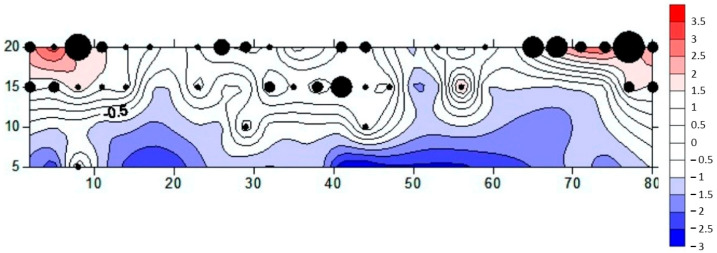
Graphical representation of the incidence and intensity of the attacks by *X. germanus* on grapevines in the studied field. The *X* and *Y* axes represent the dimensions of the vineyard, in meters. Black circles show the position and intensity of the infestation, while the red color indicates the aggregation areas where the 3 × 3 plots with the highest rate of infestation were concentrated. The blue areas indicate those parts of the vineyard where no pattern of aggregation was identified.

In total, 70 out of 809 grapevines were attacked by *X. germanus* (8.7% of the total). The majority of the infested plants were concentrated on the west side of the field, close to a concrete wall delimiting the property. In this area, considering only the first five rows of vines, the infestation rate was 16%, with 13.4 attacked plants per row on average. The intensity of the attacks and their spatial distribution are illustrated in Figure 5.

### 3.2. Curculionidae Scolytinae Attacking Vitaceae

A literature review allowed for the identification of 34 species of Scolytinae able to infest and develop on Vitaceae. Xyleborini is the most represented tribe, with 7 genera and 14 species (Table 1); Corthylini is the second most represented group with 3 genera, while *Hypothenemus* (Trypophloeini) includes the highest number of species attacking Vitaceae (11). Among Vitaceae, *Vitis* is the host genus with the highest number of Scolytinae, with 30 species out 34; *Vitis vinifera* is the species representing the majority of scolytine with 11 species: 3 Trypophloeini (*Hypothenemus carbonarius*, *H. eruditus* and *H. hampei*); 1 Dryocoetini (*Xylocleptes bispinus*); 1 Hypoborini (*Hypoborus ficus*); 5 Xyleborini (*Anisandrus dispar*, *Xyleborinus saxesenii*, *Xylosandrus. crassiusculus*, *X. discolor* and *X. germanus*).

#### 3.2.1. Bothrosternini Tribe

1. *Cnesinus elegans* Blandford, 1896

Distribution: [43]

**NTR**: **CA** GU, HO[*FM*], PA[*CH*]/**MX** CP, HI, OA, PU, TB, VC/**SA** COL[*SA*], BR[*SC*], VE.

Host plant in Vitaceae: *Vitis* sp. [44,45]—Polyphagous

#### 3.2.2. Corthylini Tribe

2. *Cryptocarenus heveae* (Hagedorn, 1912)

Distribution: [43,46,47,48]

**iAFR**: GH, DRC.

**NEA**: **US** FL.

**NTR**: **CA** CR[*PT*], HO[*AT*, *EP*], PA[*CZ*, *PM*]/**MX** CMP, CL, JA, OA, QR, TB, VC/**SA** AR[*BA*, *SE*, *TM*], BR[*AZ*, *ES*, *MG*, *MGS*, *PN*, *RJ*, *SC*, *SP*], COL[*VC*], EC[*LP*], FG, PE[*JU*, *MD*],TO, TR, VE[*BA*, *ME*]/**WI** BA, CU, CY[*CB*, *GC*], DO, DR, GR, GL, JM, MA, MO, NA[*CU*], PR, SL, VI(BR)[*GU*, *TO*, *VG*], VI(US)[*BI*, *SC*, *SJ*, *ST*].

Host plant in Vitaceae: *Vitis* sp. [44]—Oligophagous

3. *Cryptocarenus seriatus* Eggers, 1933

Distribution: [43]

**NEA**: **US** FL, TX.

**NTR**: **CA** CR, GU[*PT*], HO[*EP*], PN[*CC, PM*]/**MX** CMP, CL, JA, NA, OA, SI, TB, TM, VC/SA AR, BO[*CB*], BR[*ES*, *MGS*, *PN*, *PE*, *RGS*, *SP*], COL[*CA*, *VC*], FG, PE[*JU*], VE[*AR*, *BA*, *ME*, *ZU*]/**WI** ANQ, BA[*AD*], BR, CY[*GC*], CU, DO, GR, GL, JM, MA, MO, PR, SL, VI(BR)[*GI*], VI(US)[*BI*, *SC*].

Host plant in Vitaceae: *Vitis rotundifolia* Michx. [49]—Polyphagous

4. *Microcorthylus demissus* Wood, 1973

Distribution: [43]

**NTR**: **CA** CR/**MX** CP, OA, PU.

Host plant in Vitaceae: *Vitis* sp. [45,50]—Polyphagous

5. *Monarthrum fasciatum* (Say, 1826)

Distribution: [43]

**NEA**: **CN** ON/**US** AL, AR, CT, DE, DC, FL, GA, IL, IN, IA, KS, KY, LA, ME, MD, MA, MI, MS, MO, NE, NJ, NY, NC, OH, OK, iOR, PA, RI, SC, TN, TX, VT, VA, WV, WI.

**NTR**: MX CP.

Host plant in Vitaceae: *Vitis* sp. [50]—Polyphagous

#### 3.2.3. Cryphalini Tribe

6. *Cryphalus felis* Wood, 1989

Distribution: [43,51]

**ORI**: **ID** UP.

Host plant in Vitaceae: *Vitis* sp. [44]—Oligophagous

#### 3.2.4. Dryocoetini Tribe

7. *Xylocleptes bispinus* (Duftschmid, 1825)

Distribution: [43]

**PAL**: **AS** TK/**EU** AU, BI[*ML*], BE, BU, CR, CZ, DE, FR, GB, GE, GR, HU, IT, MC, NL, PL, RO, SK, SL, SP, SZ, UK[*CRI*, *KHM*, *ZAK*], YU/**NA** AG, EG, LB, MO, TU/**RU** ST, DAG.

Host plant: *Vitis vinifera* L. (as *V. sylvestris*) [44,52,53]—Oligophagous

#### 3.2.5. Hypoborini Tribe

8. *Hypoborus ficus* Erichson, 1836

Distribution: [43]

**iATL**: AZ.

**INO**: MG. 

**PAL**: **AS** SY, IR[*FA*, *GU*, *KH*, *TE*], IQ, IS, JO, TK [*AD*, *IS*, *ME*, *SK*]/**EU** AB, AU, BH, BU, CR, FR, GR, HU, IT, MA, MC, PT, SL, SP, SZ, UK [*CRI*], YU/**NA** AG, CANI, EG, MO, MA, TU/**RU** ST.

Host plant in Vitaceae: *Vitis* sp. [46], *Vitis vinifera* L. [54,55]

#### 3.2.6. Micracidini Tribe

9. *Micracisella nanula* (LeConte, 1876)

Distribution: [43]

**NEA**: **US** AL, FL, GA, KY, LA, MS, MO, NC, OK, SC, TX, VA. 

**NTR**: **WI** BA[AB, AN, EL, SB].

Host plant in Vitaceae: *Vitis* sp. [44]—Polyphagous

#### 3.2.7. Micracidini Tribe

10. *Hypothenemus birmanus* (Eichhoff, 1878)

Distribution: [43,56,57,58]

**iAFR**: SA.

**AUS**: **AS** QU.

**INO**: **MG** FI/**SY**.

**NEA**: **US** FL, TX.

**NTR**: **CA** CR[*PT*], HO[*AT*, *EP*], PA[*CZ*, *LS*, *PM*]/**MX** CL, JA, TB, VC, YU/**SA** BR[*BA*, *MGS*, *PE*, *SP*], TR, TO/**WI** CU, JM, PR. 

**ORI**: **BA**/**ID** AN, MP, UP/**IN** JV, SM, SU/**MA** SA/**MY**/**PH** BA, MI, MN/**SL**/**TH**/**VN** “Tonkin”.

**PAC**: **BI**/**GA**[*ES*, *FL*, *FR*, *IS*, *SCZ*]/**HA**[*HW*, *KA*, *LA*, *MA*, *MO*, *OA*]/**ME** FI, NB, NC, NG, SO[*RN*]/**MI** GU, MAR[PI]/**PO** CKI[*NU*], MQ, SOC[*TH*], SM, TO. 

**PAL**: **AS** JA[*RY*], PK, TA/**CH** GUI, GUX, HKG, HUN, SCH, YUN.

Host plant in Vitaceae: *Vitis* sp. [44]—Polyphagous

11. *Hypothenemus carbonarius* Eggers, 1943

Distribution: [43]

**AFR**: MZ, SA.

Host plant in Vitaceae: *Vitis vinifera* L. [59]—Oligophagous

12. *Hypothenemus dissimilis* (Zimmermann, 1868)

Distribution: [43,60,61]

**NEA**: **US** AL, AR, CT, DC, DE, FL, GA, IL, IN, KS, KY, LA, MD, MA, MI, MN, MS, MO, NJ, NY, NC, NH, OH, OK, PA, SC, TN, TX, VA, WV.

**iORI**: **IN** JV (possible misidentification)

Host plant: *Vitis* sp. [44]—Polyphagous

13. *Hypothenemus erectus* LeConte, 1885

Distribution: [43]

**NEA**: **US** FL, LA, TX.

**NTR**: **CA** GU[*IZ*], HO[*AT*, *EP*, *FM*, *OL*], NI[*ZE*], PA[*PM*]/**MX** CM, CP, CL, HI, JA, MC, NA, NL, OA, PU, QE, QR, SLP, SI, TB, TM, VC, YU/SA COL[*VC*], VE[*BA*, *ME*]/**WI** BA[*AN*, *GI*, *NP*], BR, CU, CY[*CB*, *GC*], DO, DR, GR, GU, JM, MA, MO, PR, SL, SV, VI(US)[*ST*].

**PAL**: **AS** TK/**CH** ANH, GAN, GUI, HEB, HEN, HUB, JIA, LIA, SCH, SHA, SHN, SHX, YUN.

Host plant in Vitaceae: *Vitis* sp. [45]—Polyphagous

14. *Hypothenemus eruditus* Westwood, 1836

Distribution: [43,58]

**AFR**: AN, BI, CI, CM, CR, DRC, GA, GH, GU, LI, NG, SL, SA, TA, TO, UG.

**iATL**: AZ.

**AUS**: **AS** NSW, QU.

**INO**: **COM**/**MC**[*MR*]/**MG** AN, AT, TM, TO/**SY**.

**NEA**: **CN** ON/**US** AL, AR, CA, DC, DE, FL, GA, IL, IN, LA, MD, MA, MI, MN, MS, NE, NH, NJ, NY, NC, OK, PA, SC, SD, TN, TX, VA, WV.

**NTR**: **CA** CR[*HE*, *IC*, *LI*, *PT*], ES[*SS*], GU[*AV*], HO[*AT*, *CR*, *EP*, *FM*, *OL*, *YO*], PA[*CZ*, *CN*, *PM*]/**MX** BCS, CM, CP, CL, GR, JA, MC, MR, NA, OA, PU, QR, SLP, SI, TB, VC, YU/**SA** AR[*BA*, *CR*, *SE*, *TM*], BR[*AM*, *BA*, *CE*, *GO*, *MGS*, *PB*, *PE*, *RGS*, *SC*, *SP*], COL, EC[*NA*], PE[*CU*, *HU*, *JU*, *LO*], TO, TR, VE[*LA*]/**WI** AN, BA[*AN*, *GA*, *GI*], BR, CU, CY[*GC*], DO, DR, GR, GL, JM, MA, MO, NV, NA[*SB*], PR, SL, SV, VI(BR)[*GU*], VI(US)[*BI*, *SC*, *SJ*].

**ORI: ID** AN, AS, PJ, UP/**IN** JV, SM/**MA** KL, SE/**MY**/**PH** MI, MN/**SL**/**TH**/**VN**.

**PAC**: **GA**[ES, FL, IS, MA, PI, RA, SC, SCZ, SF, SY]/**HA**[HW, KA, MA, OA]/**ME** FI, NC/**MI** GU/**PO** CKI[NU], MQ, TO. 

**PAL**: **AS** IR[*GU*, *NP*, *TE*], iIS, JA[*RI*], SK, iTA, iTR[*AY*, *ME*, *SM*], UK/**CH** FUJ, GUA, GUI, GUX, HEB, HUN, SCH, SHN, YUN/**iEU** AB, iCR, iFR, GB, GG, iIT, MA, iSP, UK [*CRI*]/**iNA** AG, CANI, EG, MO, MA/**RU** ST, DAG.

Host plant in Vitaceae: *Vitis* sp. [50], *Vitis vinifera* L. [44,54], *Vitis tiliifolia* Humb. & Bonpl. Ex Schult. [62]—Polyphagous

15. *Hypothenemus hampei* (Ferrari, 1867)

Distribution: [43,63,64,65]

**AFR**: AN, BF, BI, CAR, CI, CH, CR, CM, DRC, EG, ET, GA, GH, GU, KE, LI, NG, STP, SL, SA, SD, TA, TO, UG.

**iNTR**: **CA** CR[*LI*], ES, GU, HO[*CR*], NI, PA/MX CP, GR, MR, OA, PU, VC/SA BO, BR, COL, EC, PE, SU/**WI** CU, DR, JM, MA, PR.

**iORI**: ID/IN JV, SM/LA/MA/PH/SL/TH/VN.

**iPAC**: **HA**[*HW, LA*]/**ME** FI, NC/**MI** CLI[*POH*], MAR/**PO** SOC[*TH*], SM.

**iPAL**: **AS** IR, TA/**CN** HAI/**NA** CANI.

Host plant in Vitaceae: *Tetrastigma lanceolarium* (Roxb.) Planch. (as *Vitis*) [66], *Vitis* sp. [67], *Vitis vinifera* L. [54]—Oligophagous

16. *Hypothenemus interstitialis* (Hopkins, 1915)

Distribution: [43,53]

**NEA**: **US** AL, AR, CT, DC, FL, GA, IL, IN, KS, KY, LA, MD, MA, MS, MO, NJ, NC, OK, PA, SC, TN, TX, VA, WV. 

**NTR**: **CA** BE[*OW*], CR[*HE*, *SJ*], GU[*BV*, *ES*], HO[*EP*, *FM*], PA[*CN*, *PM*]/**MX** CM, CP, GR, JA, NA, OA, QR, TB, VC, YU/**SA** BR[*MG*, *MGS*, *PA*, *RJ*, *RD*, *SP*], COL[*SA*], PE[*MD*], TR/**WI** CU, DR, GR, JM, NA[*SA*], PR.

Host plant in Vitaceae: *Vitis* sp. [44]—Polyphagous

17. *Hypothenemus javanus* (Eggers, 1908)

Distribution: [43,57]

**AFR**: CI, CM, CR, DRC, GA, GH, MZ, SL, STP, ZA.

**NEA**: **US** FL.

**NTR**: **CA** PA[PM]/**MX** JA, VC/**SA** BR[*AM*, *CE*, *GO*, *MG*, *MGS*, *MGE*, *PA*, *PN*, *RJ*, *RGS*, RD, *SP*, *TC*], VE[*MI*]/**WI** AN, BA[*AN*, *GA*], CU, CY[*CB*, *GC*], DO, DR, GL, JM, MO, NA[*CU*], NV, PR, SL, SV, VI(BR)[*ANQ*, *GU*, *TO*], VI(US)[*SC*, *SJ*, *ST*].

**ORI**: “Borneo”/**ID** AN, AS, KE,/**IN** JV/**MA**/**PH** LU/SL/TH.

**PAL**: **AS** JA, TA/**CH** GUI, GUX, HAI, HUB, HUN, SCH, YUN.

Host plant in Vitaceae: *Vitis* sp. [44]—Polyphagous

18. *Hypothenemus obscurus* (Fabricius, 1801)

Distribution: [43]

**AFR**: AN, CI, CR, DRC, EG, GH, LI, MZ, NG, RW, SL, SA, TA, UG. 

**AUS**: AS. 

**INO**: **MG** AN, AT, FI/**SY**[*LDI*]. 

**NEA**: **US** AL, AR, DE, FL, GA, KY, LA, MS, NC, OK, PA, SC, TX, VA, WV. 

**NTR**: **CA** CR[*SJ*], ES[*LL*], GU, HO[*EP*, *FM*, *OL*, *YO*], PA[*CZ*, *PM*]/**MX** CP, JA, NA, OA, PU, SLP, SO, TB, TM, VC, YU/**SA** AR[*SE*], BR[*AZ*, *CE*, *GO*, *MGS*, *PA*, *RJ*, *RGS*, *SC*, *SP*], COL[*AN*, *SA*, *VC*], EC, GY, PE[*MD*], PG, SU, TR, VE[*AR*, *BA*, *BO*, *ME*, *ZU*]/**WI** AN, BA[*AD*, *GI*, *SB*], BR, CY[*CB*, *LC*], CU, DO, DR, GR, GL, HA, JM, MA, MO, NA[*CU*, *SA*], NV, PR, SL, SV, TC, VI(BR)[*GU*, *TO*], VI(US)[*BI*, *SC*, *SJ*, *ST*].

**ORI**: **IN** JV/**PH** LU/**SL**.

**PAC**: **GA**[*DW*, *ES*, *FL*, *GE*, *IS*, *SA*, *SC*, *SCZ*]/**HA**[HW, KA, OA]/**ME** FI/**MI**/**PO** CKI[NU], SM, TO.

**PAL**: **AS** TA, TK/**iEU** GB/**NA** EG.

Host plant in Vitaceae: *Vitis* sp. [68]

19. *Hypothenemus squamosus* (Hopkins, 1915)

Distribution: [43]

**NEA**: **US** FL, TX.

**NTR**: **MX** CM, CL, JA, QR, TM, VC, YU/**SA** TO/**WI** BA[*SB*], CU, PR.

Host plant in Vitaceae: *Parthenocissus quinquefolia* (L.) Planch. [44]—Polyphagous

20. *Hypothenemus vitis* Browne, 1970

Distribution: [43]

**AFR**: SA. 

Host plant in Vitaceae: *Vitis* sp. [44]—Monophagous

#### 3.2.8. Xyleborini Tribe

21. *Anisandrus apicalis* (Blandford, 1894)

Distribution: [43,69]

**ORI**: **ID** MG, SK, WB/**MY**/**TH** CM. 

**PAL**: **AS** BT, JA, NK, NP[*DH*, *GA*, *KO*], SK, TA/**CH** ANH, GUI, GUX, HAI, JIX, SCH, SHX, XIZ, YUN/**RU** FE[*KI]*. 

Host plant in Vitaceae: *Vitis* sp. [44]—Polyphagous

22. *Anisandrus dispar* (Fabriscius, 1792)

Distribution: [43]

**iNEA**: **CA** BC, NB, NS, ON/**US** CA, DC, FL, ID, IL, IN, ME, MD, MA, MI, MT, NV, NH, NJ, NY, NC, OH, OR, PA, RI, UT, VT, VA, WA, NV, WI.

**ORI**: ID.

**PAL**: **AS** IR[CB, GO, GU, HA, IS, KH, MA, MR, TE, ZA], JA, KZ, MG, NK, SK, TK [AM, AN, AR, BA, BO, BU, CO, DE, DU, GI, GU, HA, IS, KR, KS, MU, NI, OR, RI, SM, TR, SK, SM, TR, WM, ZO]/EU AB, AU, BE, BH, BU, BY, CR, CT, CZ, DE, EN, FI, FR, GB, GE, GR, HU, IT, LA, LT, MC, MD, NL, NR, PL, SK, SL, SP, ST, SV, SZ, UK [ÈER, ÈNG, CRI, ÈRK, DON, IFR, KHE, KHM, KHR, KIR, KYI, LUG, LWI, MYK, ODE, SUM, TER, VOL, ZAK, ZAP], YU, “Caucasus”/**CH** HEI, SHA/**RU** ES, FE, NT, WS, CFD, DAG, SKR. 

Host plant in Vitaceae: *Vitis* sp. [70], *Vitis vinifera* L. [46,71,72]—Polyphagous

23. *Cnestus mutilatus* (Blandford, 1894)

Distribution: [43,69]

**INO**: ANI.

**iNEA**: **US** AL, AR, DE, FL, GA, IL, IN, KY, LA, MD, MS, MO, NJ, NC, OH, PA, SC, TN, TX, VA, WV.

**ORI**: “Borneo”/**ID** AN, AS/**IN** BI, JV, SM/**MA**/**MY**/**SL**/**TH** CM/**VN** CB.

**PAC**: **ME** NG.

**iPAL**: **AS** JA[*KY, RI*], KO, SK, TA/CH ANH, FUJ, GUI, HAI, HKG, JIA, JIX, SCH, SHA, SHG, YUN, ZHE/**RU**. 

Host plant in Vitaceae: *Vitis rotundifolia* Michx. [73,74]—Polyphagous

24. *Euwallacea piceus* (Motschulsky, 1863)

Distribution: [43]

**AFR**: AN, CI, CM, DRC, EG, GH, KE, NG, SA, TA, UG.

**AUS**: AS QU.

**INO**: **MG** AN, AT, TM.

**ORI**: **ID** AN, SK/**IN** JV, ME/**MA** SA, SB/**PH** LU, MI, PA/**SL/TH** CM, NST, PT.

**PAC**: **ME** BI, FI, SO, PNG[*MA, OR, WS*], VA/**PO** SM. 

**PAL**: **AS** KO, TA. 

Host plant in Vitaceae: *Vitis discolor* [44]—Polyphagous

25. *Euwallacea xanthopus* (Eichhoff, 1868)

Distribution: [43]

**AFR**: CI, DRC, KE, NG, SA[*NA*], TA, UG.

Note: All the records from Asia and the Pacific region given in Bright [44] should be attributed to *Euwallacea semirudis* (Blandford, 1896) 

Host plant in Vitaceae: *Rhoicissus erythrodes* (Fresen.) Planch. [44]—Polyphagous

26. *Premnobius cavipennis* Eichhoff, 1878

Distribution: [43]

**AFR**: AN, BUR, CI, CM, CR, DRC, EG, ET, GA, GB, GH, GU, KE, ML, MU, MZ, NA, NG, RW, SE, SO, SA, TA, UG, ZA, ZI. 

**INO**: **MC**[*MR*]/**MG** AT, TM.

**NEA**: US FL.

**NTR**: **CA** BE[*CY*], CR[*PT*], HO[*AT*, *CR*, *OL*], PA[*CN*, *PM*]/**MX** CM, CP, GR, JA, MR, OA, QR, TB, TM, VC, YU/**SA** BR[*BA*, *ES*, *RGS*, *SP*], COL[*HU*], EC[*CP*, *LR*, *VL*], FG, GY, PE[*JU*, *LO*, *MD*], SU, TR, VE[*BA*, *ME*]/**WI** BA[*AD*, *GB*, *NP*], BR, CU, DO, DR, GL, HA, JM, MA, MO, PR, SK, SL, VI(BR)[*TO*], VI(US)[*SC*, *SJ*, *ST*].

Host plant in Vitaceae: *Vitis* sp. [44]—Polyphagous

27. *Xyleborinus andrewesi* (Blandford, 1896)

Distribution: [43]

**AUS**: NZ.

**AFR**: GB, KE, ZA.

**PAC**: **HA** HW?/**ME** PNG[*MA*, *OR*, *SI*, *WS*]/**NZ**.

**INO**: SY.

**iNEA**: **US** FL.

**iNTR**: **WI** CU, JM.

**ORI**: **BA**/**ID** AN, AS, BI, KN, MP, MA, SK, TN, UP, WB/**IN** JV/**MA** SB/**MY**/**PH**/**SL**/**TH** CH, CM, CP, KA, KP, LO, MHS, NA, NN, NR, NST, PT, SN, ST, SU/**VN**.

**PAC**: **iHA**[*HW*, *KA*, *OA*]/**MI**.

**PAL**: **AS** JA, NP[NA], TA/**CH** YUN. 

Host plant in Vitaceae: *Leea* sp. [44]—Polyphagous

28. *Xyleborinus saxesenii* (Ratzeburg, 1834)

Distribution: [43]

**iAFR**: CM, NG, SA.

**iATL**: AZ. 

**AUS**: **AS** QU/**NZ**. 

**iNEA**: **CN** BC, NB, NS, ON, QC/**US** AL, AZ, AR, CA, CO, CT, DE, DC, FL, GA, ID, IL, IN, IA, KS, KY, LA, ME, MD, MA, MI, MS, MO, NE, NV, NH, NJ, NM, NY, NC, OH, OK, OR, PA, RI, SC, SD, TN, TX, UT, VT, VA, WA, WV, WI.

**iNTR**: **MX** BCN, BCS, GT, HI, NL/**SA** AR[BA, CH, ER, SA, TM], BR[ES, SC, MGS, PN, SC, SP], CH[AR, BB, LL, LR, ML, OH, VP], EC, PG[GU], UR[DU, PA, RN, RO, TA]. 

**ORI**: **ID** AS, JK, SK, UP, WB/**PH/VN**.

**PAC**: **BI/GA**[*IS*, *SA*, *SCZ*]/**iHA**[*HW*, *KA*, *MA*, *MO*, *OA*]/**ME** NC, NG/**PO** SM.

**PAL**: **AS** IR, IS, JA, KI, MG, NK, SK, SY, TA, TD, TM, TK[*AM*, *AN*, *AR*, *BO*, *DU*, *GI*, *HA*, *IP*, *IS*, *KO*, *KN*, *ME*, *MU*, *OR*, *RI*, *SK*, *SM*, *SI*, *TR*, *ZO*]/**CH** ANH, FUJ, GUI, GUX, HEB, HEI, HUN, JIA, JIL, JIX, NE, NIN, SCH, SHA, SHX, XIZ, YUN, ZHE/**iEU** AB, AL, AU, BE, BI[*ML*], BU, BY, CR, CZ, DE, EN, FR, GB, GE, GR, HU, IT, LA, LT, LU, KZ, MA, MC, MD, NL, NR, PL, PT, RO, SL, SP, SV, SZ, UK [*ÈER*, *ÈNG*, *CRI*, *DON*, *IFR*, *KHE*, *KHM*, *KHR*, *KIR*, *KYI*, *LUG*, *LWI*, *MYK*, *ODE*, *SUM*, *TER*, *VOL*, *ZAK*], YU, “Caucasus”/**NA** AG, CANI, EG, LB, MO, MA, TU/**RU** CT, ES, FEFD[*KA*, *KI*, *SKI*], NT, ST, WS, DAG, SKR, SBFD.

Host plant in Vitaceae: *Vitis* sp. [46], *Vitis vinifera* L. [72,75,76]—Polyphagous

29. *Xyleborus principalis* Eichhoff, 1878

Distribution: [43,77] 

**AFR**: CM, DRC, EG, GA, GU, KE, SL, TA, UG.

**INO**: MG. 

Host plant in Vitaceae: *Rhoicissus erythrodes* (Fresen.) Planch. [44]—Polyphagous

30. *Xylosandrus compactus* (Eichhoff, 1815)

Distribution: [43,78,79]

**AFR**: BI, CI, CM, CM[*GC*], EG, GA, GH, LI, MU, NG, SE, SL, SA, TA, UG.

**INO**: COM[*GC*]/MC[*MR*, *RÉ*]/MG AT, FI, TM/SY.

**iNEA**: **US** AL, AK, FL, GA, IL, IN, KY, LA, MS, NC, PA, SC, TN, TX.

**iNTR**: **CA** PA/**SA** BR[*AZ*, *CE*, *ES*, *MG*, *PN*, *RJ*, *RD*], FG, PE[*CU*, *LO*, *MD*], TR/**WI** BR, CU, CY[*GC*], DO, DR, GR, GL, MA, MO, NA[*SA*], PR, SL, SV, VI(BR)[*GU*, *TO*, *VG*], VI(US)[*ST*].

**ORI**: **ID** TN/**IN** JV, SM, SU/**MA** SB/**PH**/**SL**/**TH** CH, CM, CP, KK, NN, NR, NST, SO, ST, TR/**VN** “Tonkin”.

**PAC**: **BI**/**iHA** [*HW*, *KA*, *LA*, *MA*, *MO*, *OA*]/**ME** FI, NC?, PNG[*MA*]/**PO** SM.

**PAL**: **AS** JA, JA[*RI*], TA/**CH** FUJ, GUA, GUI, GUX, HAI, HUB, HUN, SCH, YUN, ZHE/**iEU** FR, GR, IT, SP. 

Host plant in Vitaceae: *Vitis* sp. [44], *Vitis labruscana* L.H. Bailey (*Vitis labrusca L.* × *Vitis vinifera* L.) [80,81], *Vitis labrusca L.* [82]—Polyphagous

31. *Xylosandrus crassiusculus* (Motschulsky, 1866)

Distribution: [43]

**AFR**: BI, CI, CM, CR, DRC, GA, GH, EG, KE, MU, NG, SL, TA.

**INO**: **MC**[*MR*]/**MG** AN, AT, TM, TO/**SY**[*MI, SI*].

**iNEA**: **CN** ON/**US** AL, AR, CT, CO, DE, FL, GA, IL, IN, KS, KY, LA, MD, MA, MI, MS, MO, NE, NJ, NY, NC, OH, OK, OR, PA, RI, SC, TN, TX, VA, WV.

**iNTR**: **CA** CR[*AL*, *HE*, *LI*, *PT*], GU[*IZ*], PA[*CN*, *PM*]/**SA** AR[*BA*, *MN*, *TM*], BR[*AM*, *PE*, *RJ*, *SP*], FG, UR[*PA*, *RV*, *SJ*]/**WI** BA[*NP*], PR.

**ORI**: **ID** AN, AS, HP, MA, MP, TN, UP, WB/**IN** JV, SM, SU/**MA** SA, SB, SE/**MY**/**PH** LU, MI/**SL**/**TH** (all regions)/**VN**.

**PAC**: **BI**/**iHA** [*HW*, *KA*, *MA*, *MO*, *OA*]/**ME** NC, PA, PNG [*MA*], SO/**MI** GU/**PO** SM.

**PAL**: **AS** BT, JA[*KT*, *KY*], NK, NP[*BH*, *DH*, *GA*, *KO*, *ME*, *NA*], TA/**CH** ANH, FUJ, GUA, GUI, HKG, HAI, HEB, HUB, HUN, SCH, SHA, SHN, XIZ, YUN, ZHE/**iEU** GE, IT, SL, SP. 

Host plant in Vitaceae: *Leea asiatica* (L.) Ridsdale (as *L. crispa*), *Leea sambucina* (ambiguous taxon) [44], *Vitis* sp. [46], *Vitis vinifera* L. [83,84]—Polyphagous

32. *Xylosandrus discolor* (Blandford, 1898)

Distribution: [43]

**AUS**: **AS** QU. 

**ORI**: **ID** AN, AS, SK, TN, UP/**IN** JV/**MA**/**MY**/**SL**/**TH** (all regions)/**VN** TQ, YB PAC: ME PA, PNG[MA]/**MI**?

**PAL**: **AS** JA[*RI*], TA/**CH** FUJ, GUA, HAI, SCH, YUN. 

Host plant in Vitaceae: *Vitis vinifera* L. [44]—Polyphagous

33. *Xylosandrus germanus* (Blandford, 1894)

Distribution: [43]

**iNEA**: **CN** BC, NS, ON, QC/**US** AL, AR, CT, DE, FL, GA, IL, IN, KS, KY, LA, ME, MD, MA, MI, MS, MO, NH, NJ, NY, NC, OH, OR, PA, RI, SC, TN, TX, VT, VA, WA, WV, WI. 

**ORI**: **TH**[*CM*]/**VN** LC.

**iPAC**: **HA** OA.

**PAL**: **AS** JA[*HO*, *KT*, *KY*, *RI*], NK, SK, TA, TK[*DU*, *OR*, *SM*]/**iEU** AU, BE, CR, CZ, DE, FR, GB, GE, GG, HU, IT, NL, PL, SI, SP, SZ, UK/**CH** ANH, FUJ, GUA, GUI, GUX, HAI, HEN, HUB, HUN, SCH, SHA, SHX, XIZ, YUN, ZHE/**iRU** FEFD[*KI*, *PK*], ST. 

Host plant in Vitaceae: *Ampelopsis brevipedunculata* (Maxim.) Trautv. (as var. *heterophilla*) [71], *Vitis* sp. [20,44,46], *Vitis coignetiae* Pulliat ex Planch. [20,85], *Vitis vinifera* L. [30,39,85]—Polyphagous.

34. *Xylosandrus morigerus* (Blandford, 1894)

Distribution: [43]

**iAFR**: DRC, GA.

**AUS**: **AS** QU.

**INO**: **MC**[*MR*, *RÉ*]/**MG**.

**iNEA**: **US** NJ.

**iNTR**: **CA** CR[*LI*, *PT*, *SJ*], HO, NI, PA[*CH*, *CZ*, *PM*]/**MX** CM, CP, OA, TB, VC/**SA** BR, COL[*AN*, *VC*], EC[*CP*, *LR*, *NA*, *VL*], VE [*BA*, *ME*, *MI*]/**WI** GL, MA, PR.

**ORI**: **ID** AS, KN, SK, TN, WB/**IN** JV, SM, SU/**MA** KE, SA, SB/**PH** LU/**SL**/**TH** CH, CM, CY, NST, SO, ST, TR/**VN** “Tonkin”.

**iPAC**: **GA** SCZ/**HA** [*HW*]/**ME** BI, FI[*VIL*], PNG[*MA*, *OR*, *WS*], SO[**GC**]/**MI** CLI, GU, MAR[*TI*]/**PO** CKI[*RT*?], SM[*UP*], TO.

**iPAL**: **AS** JO, LN, TA/**EU** AU, FR, GB, IT. 

Host plant in Vitaceae: *Vitis* sp. [44]—Polyphagous

Dubious attribution: *Euwallacea fornicatus* species complex

The *Euwallacea fornicatus* species complex comprises seven species from Asia and Oceania: *E. fornicatus* (Eichhoff, 1868), *E. fornicatior* (Eggers, 1923), *E. whitfordiodendrus* (Schedl, 1942), *E. schultzei* (Schedl, 1951), *E. perbrevis* (Schedl, 1951b), *E. tapatapaoensis* (Schedl, 1951) and *E. kuroshio* Gomez and Hulcr, 2018 [86]. Since these species were grouped into one species in the past and their identification remains challenging [86], the association of a particular species with its host often remains difficult and uncertain. Consequently, the record of *Vitis vinifera* indicated by Eskalen et al. [87] and Gomez et al. [88] remains difficult to attribute.

## 4. Discussion

### 4.1. Xylosandrus germanus Infestation on V. vinifera

Given the peculiar pattern of attack that characterized our case study (i.e., attacks more concentrated on the grape plants close to the concrete wall and in the most depressed area of the vineyard), it is plausible that the major factor that determined the spatial position of the *X. germanus* infestations was stress due to flooding. The majority of the infested plants were, in fact, located in the most depressed part of the field (Figure 5), where it was more plausible that excess moisture had accumulated due to the heavy rains of the previous weeks. Furthermore, the concrete wall delimiting the field might have played a key role in stressing the rows of grapevines in its vicinity, working as physical barrier that probably limited the normal flow of rainwater and dispersion of residual moisture. *Xylosandrus germanus* is notoriously known to attack plants subjected to flood stress [89,90] and it is particularly attracted by ethanol, one of the most common volatiles released by stressed plants [91,92,93], including *V. vinifera* [94].

We did not observe any peculiar analogy with the infestation cases that occurred on *Vitis* in Germany [39] and China [30], possibly suggesting that the colonization of grapevines is regulated by a multiple-factors mechanism substantially depending on the environmental and agricultural conditions.

### 4.2. Scolytinae on Vitaceae

Vitaceae, and *Vitis* spp., in particular, host a relatively low number of Scolytinae worldwide; however, it is interesting to note how a great proportion of these species are widely distributed, naturally occurring or introduced on almost all continents. The majority of the taxa considered are polyphagous, possibly attacking Vitaceae sporadically or only under certain conditions. Furthermore, the association between some species of Scolytinae and the Vitaceae still remains to be clarified, especially in regard to poorly known species and species whose records were sporadic and never reconfirmed.

However, Xyleborini members must be considered species of primary phytosanitary and economic interest as they are already present (also as invasive) in the areas suited to viticulture and already documented as pests of *Vitis* spp. (see above).

## 5. Conclusions

Sustainable production remains one of the major challenges in modern viticulture, especially in regard to pest management [95]. In an ever-changing world increasingly subject to climate change and human-assisted movements of exotic fauna, understanding the association between the incidence of attacks on grapevines and the emergence of wood-boring pests becomes of primary importance. Extreme weather events such as those that have characterized recent years (heavy rains, sudden frosts and prolonged drought) may predispose *V. vinifera*, an already susceptible plant, to further stressful conditions and consequently make it more attractive to pests, including woodborers. What we have reported here may not just be a solitary case, but could be a first warning to be considered in the phytosanitary management of vineyards. *Xylosandrus germanus* is widespread in Italy and the rest of Europe, and it is plausible that, in a territory densely subject to viticulture and close to forested areas, such as our study area, it may become a constant presence in our vineyards in the near future. Furthermore, other invasive species such as *Xylosandrus crassiusculus* deserve just as much attention.

## Figures and Tables

**Table 1:** Tribes and genera of Scolytinae developing on Vitaceae worldwide.

Tribe	Genera	No. Spec. on Vitaceae	No. Spec. on *Vitis* sp.
Bothrosternini Blandford, 1896	*Cnesinus* LeConte, 1868	1	1
Corthylini LeConte, 1876	*Microcorthylus* Ferrari, 1867	1	1
	*Monarthrum* Kirsch, 1866	1	1
	*Cryptocarenus* Eggers, 1937	2	2
Cryphalini Lindemann, 1877	*Cryphalus* Erichson, 1836	1	1
Dryocoetini Lindemann, 1877	*Xylocleptes* Ferrari, 1867	1	1
Hypoborini Nusslin, 1912	*Hypoborus* Erichson, 1836	1	1
Micracidini LeConte, 1876	*Micracisella* Blackman, 1928	1	1
Trypophloeini Nüsslin, 1911	*Hypothenemus* Westwood, 1836	11	10
Xyleborini LeConte, 1876	*Anisandrus* Ferrari, 1867	2	2
	*Cnestus* Sampson, 1911	1	1
	*Euwallacea* Hopkins, 1915	2	1
	*Premnobius* Eichhoff, 1878	1	1
	*Xyleborinus* Reitter, 1913	2	1
	*Xyleborus* Eichhoff, 1864	1	0
	*Xylosandrus* Reitter, 1913	5	5

## Data Availability

Data available upon request.
